# Bioinformatics combined with machine learning for potential biomarker screening and immune infiltration analysis in neonatal sepsis

**DOI:** 10.3389/fped.2026.1702073

**Published:** 2026-03-31

**Authors:** Na Liu, Zhiyong Tan

**Affiliations:** 1 Department of Pediatrics, Wuhan Fourth Hospital, Wuhan, Hubei, China; 2 Informatization Centre, Wuhan Vocational College of Software and Engineering, Wuhan, Hubei, China

**Keywords:** neonatal sepsis, bioinformatics, machine learning, biomarkers, immune infiltration

## Abstract

**Objective:**

Apply bioinformatics combined with machine learning algorithms to screen potential biomarkers of neonatal sepsis (NS) and explore the correlation between biomarkers and immune cells.

**Methods:**

Expression profiling data containing NS samples and control samples were downloaded from the Gene Expression Omnibus (GEO) database. First, the differentially expressed genes (DEGs) were found. Then gene set enrichment analysis (GSEA) was carried out to reveal the significantly enriched pathways, and the core genes within these pathways were aggregated. Subsequently, weighted gene co-expression network analysis (WGCNA) was used to identify the gene modules significantly correlated with NS, from which the key genes were screened. The feature genes were selected by the use of three machine learning algorithms: least absolute shrinkage and selection operator (LASSO) regression, support vector machine recursive feature elimination (SVM-RFE), and random forest cross-validation (RF-CV), and then the potential biomarkers of NS were identified by logistic regression analyses. The dataset was randomly split into training and test sets. A logistic regression model with biomarkers was constructed using the training set, and its diagnostic value was evaluated separately on both the training and test sets. In addition, immune infiltration analysis was performed using immune cell deconvolution algorithm CIBERSORT to explore the correlation between biomarkers and immune cells.

**Results:**

Overall, after overlapping the filtered DEGs, the core genes of GSEA, and the key genes of WGCNA, we detected 14 intersecting genes. Three machine learning algorithms further selected 4 feature genes, and logistic regression analyses identified ACSL1 and CD3D as potential biomarkers of NS. The classification accuracies of the logistic regression model were 0.883 and 0.902 on the training set and test set, respectively. The area under the curve (AUC) of the receiver operating characteristic curve (ROC) was 0.961 and 0.966, and the calibration curves were close to the ideal curve. It should be noted that these AUC values were estimated within this discovery GEO cohort and require confirmation in independent external cohorts. Immune infiltration analysis showed significant changes (*P* < 0.05) in the infiltration abundance of 13 immune cell types, and a strong correlation (>0.6) existed between ACSL1, CD3D and neutrophils, naive CD4^+^ T cells, and CD8^+^ T cells.

**Conclusion:**

ACSL1, CD3D are potential biomarkers of NS and may play an important role in the pathogenesis of NS by modulating immune cell functions such as neutrophils, naive CD4^+^ T cells, and CD8^+^ T cells. These conclusions should be regarded as exploratory biomarker discovery rather than diagnostic readiness.

## 1 Introduction

Neonatal sepsis (NS) is a prevalent and severe infectious condition during the neonatal period, representing a major cause of mortality and serious complications in the neonatal intensive care unit. The global incidence of NS is 2,022 (95% CI 1,099–4,360) cases per 100,000 live births, with mortality between 11% and 19% (1). The incidence of NS is 1.8 times greater in middle-income nations and up to 3.5 times greater in low-income countries compared to high-income ones (2). In China, despite differing opinions on the evolution of NS incidence over the past thirty years (3, 4), even the optimists acknowledge that NS continues to pose a significant public health challenge, necessitating ongoing focus and effective interventions to further mitigate its disease burden (4).

The etiological agents of NS encompass bacteria, viruses, and fungi (5–7). Its clinical manifestation is often atypical, potentially presenting with symptoms such as temperature instability, poor feeding, respiratory distress, oliguria, diarrhea, jaundice, and purpura (8), thereby complicating early recognition and diagnosis of NS. Blood culture is the gold standard for diagnosing NS; however, its clinical use is constrained by a low positive detection rate, long turnaround time, and the impact of antibiotic therapy (9). Other diagnostic tests unrelated to culture, such as complete blood count, C-reactive protein, and procalcitonin, exhibit a lack of specificity and are suboptimal for diagnosing NS (10). Furthermore, while all newborns with suspected NS are advised to undergo evaluation for cerebrospinal fluid (11), the collection of cerebrospinal fluid via lumbar puncture is an invasive procedure that is frequently met with apprehension by families. Numerous studies have demonstrated that screening for extremely sensitive and specific biomarkers is crucial for diagnosing NS and may serve as a possible target for its therapy (12, 13).

High-throughput sequencing facilitates the examination of alterations in disease gene expression and the identification of possibly illness-associated genes, thereby establishing a foundation for the discovery of novel diagnostic and therapeutic strategies (14). Bioinformatics methodologies, including DEGs analysis, GSEA, and WGCNA, have demonstrated considerable efficacy in identifying biomarkers associated with disease and possible targets for therapy (15–17). Moreover, both supervised and unsupervised learning in machine learning exhibited considerable benefits in elucidating the inherent relationships within high-dimensional data (18, 19), and also displayed substantial applicability in analyzing high-dimensional transcriptome data and identifying biologically significant key genes (20, 21). This study utilized NS high-throughput sequencing data to analyze and identify potential biomarkers of NS by integrating DEGs, GSEA, WGCNA, and various machine learning algorithms. Additionally, immune infiltration analysis was conducted using the CIBERSORT algorithm to evaluate the correlation between biomarkers and immune cells, aiming to provide a foundational basis for the diagnosis and treatment of NS.

## 2 Materials and methods

### 2.1 Materials

#### 2.1.1 Data sources

The GEO is an international public database containing high-throughput gene expression and epigenomics datasets obtained by next-generation sequencing and microarray technologies (22). The GEO database was searched using the formula “NS AND Homo sapiens [Organism] AND Expression profiling by array [Filter],” and the criteria for inclusion were: the dataset contained NS samples and control samples; all samples were free of diseases other than NS; and the number of both NS samples and control samples was greater than 50. The available dataset, GSE25504, consisted of a subset of data from four different sequencing platforms (GPL570, GPL6947, GPL13667, and GPL15158), with a total of 170 samples. After excluding the samples such as suspected cases, 135 samples remained, comprising 64 NS samples and 71 control neonates.

#### 2.1.2 Data quality

**Figure 1:**
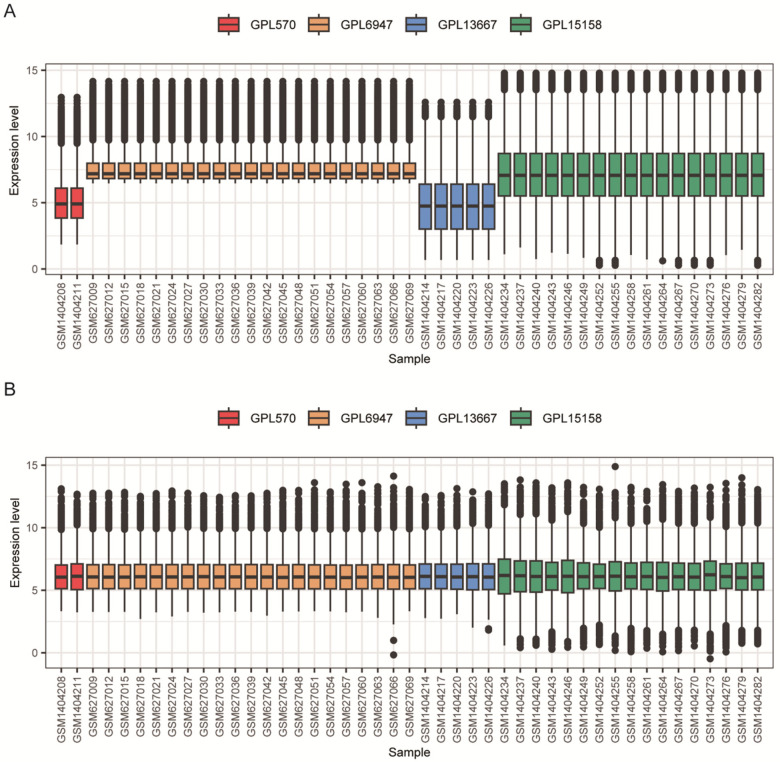
Batch effect correction normalizes the distribution of gene expression across microarray platforms in the GSE25504 dataset. **(A)** Distribution of gene expression values across samples prior to batch effect removal. Each box represents one sample, colored by its original microarray platform: GPL570 (red), GPL6947 (orange), GPL13667 (blue), and GPL15158 (green). Substantial differences in median expression and distribution ranges are evident across platforms, indicating a strong batch effect. **(B)** Distribution of gene expression values after correction for platform-specific batch effects using the “removeBatchEffect” function. The medians and interquartile ranges across all samples and platforms are successfully aligned. For visual clarity, only one-third of the total samples are displayed.

All the relevant analyses in this study were done by the R language. Because GSE25504 integrates four expression platforms (GPL570, GPL6947, GPL13667 and GPL15158), we mapped probes to official gene symbols within each platform and retained only the genes shared by all platforms, yielding 6,423 common genes. This intersection step avoids platform-specific missingness and ensures that downstream analyses (DEGs, WGCNA, GSEA, and immune deconvolution) are performed on a comparable feature space. Although 6,423 is smaller than the full transcriptome, it still represents a large subset of reliably measured genes and is sufficient for robust biomarker discovery in multi-platform microarray integration. Due to the batch effect between datasets of different platforms, see Figure 1A, only 1/3 of the samples are shown here in steps of 3, using the “removeBatchEffect” function of the “limma” package (v3.58.1) to remove the batch effect (Figure 1B).

### 2.2 Methods

#### 2.2.1 Screening of DEGs and calculation of GSEA

The gene expression data of the samples were subjected to principal component analysis (PCA) using the “FactoMineR” package (v2.11), revealing the distribution of NS samples and control samples in the principal component space. The DEGs between groups were analyzed using the “limma” package (v3.58.1), and the results of the differential analysis were shown by volcano plot. The DEGs were filtered using an adjusted *P* < 0.05 and $|\mathrm{lo}g_{2}\;\mathrm{FC}|>0.5$ as the thresholds, in which the DEGs with $\;\mathrm{lo}g_{2}\;\mathrm{FC}>0.5$ were labeled as up-regulated genes, while those with $\mathrm{lo}g_{2}\mathrm{FC}<0.5$ were labeled as down-regulated genes. We used a relatively lenient effect-size cutoff (|log_2_FC| > 0.5) together with multiple-testing control to avoid discarding biologically meaningful, moderately shifted genes in a complex systemic disease such as sepsis. Importantly, this initial DEG list was subsequently subjected to stringent multi-step filtering (WGCNA module relevance, GSEA leading-edge/core genes, and consensus selection across three machine-learning methods), implementing a “broad-in, strict-out” strategy to reduce false positives while retaining potential regulatory signals.

GSEA is a computational method used to determine whether a set of *a priori* genes exhibit statistically significant differences in consistency between two biological states. All DEGs were sorted by $\mathrm{lo}g_{2}\mathrm{FC}$ value from largest to smallest, and “c2.cp.kegg_legacy.v2023.2.Hs.symbols” from the molecular signature database (MSigDB) was used as the set of *a priori* genes. The enrichment scores for each biological pathway in the *a priori* gene set were calculated across the sorted DEGs using the “clusterProfiler” package (v4.10.1). The biological pathways were considered statistically significant when adjusted *P* < 0.05, and the core genes within these significant pathways were aggregated.

#### 2.2.2 Construction of co-expression gene modules

The gene co-expression network was constructed using the “WGCNA” package (v1.73) to identify the gene modules that are significantly related to NS, and then the key genes were selected from them. Firstly, hierarchical clustering was used to cluster all samples and eliminate outliers. Then a suitable soft threshold was selected to make the connections between genes conform to the scale-free network characteristics. After constructing the topological overlap matrix with the soft threshold, gene modules were generated by hierarchical clustering and dynamic shear tree methods. The first principal component of each module was extracted as the module eigengene (ME), and the eigengene significance (ES) of each module was calculated, then the module was considered as a key module when |ES|>0.6 and *P* < 0.05. The module membership (MM) and gene significance (GS) of genes within the key modules were calculated to screen key genes, which were defined as those with |MM|>0.8 and |GS|>0.2 (23).

#### 2.2.3 Machine learning algorithms for selecting feature genes

Take the intersection of the filtered DEGs, the core genes of GSEA, and the key genes of WGCNA. Then we selected three complementary and widely used feature-selection approaches—LASSO (embedded, sparse linear model) (24), SVM-RFE (wrapper method optimizing classification margin) (25), and Random Forest (non-linear ensemble with variable importance) (26)—to select feature genes and reduce method-specific bias. We retained only the intersection of genes selected by all three methods to obtain a conservative, high-confidence set of candidate biomarkers. The corresponding R packages for these three algorithms are, in order, “glmnet” package (v4.1.8), “e1071” package (v1.7.14), and “randomForest” package (v4.7.1.2). *λ*, which minimizes the cross-validation error, was taken as optimal in LASSO regression; a radial basis function kernel was used in SVM-RFE; for Random Forest, the final number of trees was chosen at the point where the out-of-bag (OOB) error stabilized at its minimum, and the importance of feature gene was calculated by linear scaling in RF-CV. To reduce the risk of overfitting, both SVM-RFE and RF-CV use 5-repeated 3-fold cross-validation. These three algorithms select their corresponding feature genes and then take the intersection to get the feature genes related to NS.

#### 2.2.4 Logistic regression for biomarkers identification and diagnostic value evaluation

Univariate logistic regression analysis was conducted with the following independent variables: the feature genes selected by the machine learning algorithms, and the potential influencing factors for NS, including birth weight, birth gestational age, gender, and whether antibiotics were used. Birth weight and gestational age were transformed into dichotomous variables: according to the World Health Organization (WHO) criteria (27) to determine whether the birth weight was low birth weight (birth weight < 2,500 g was low birth weight) and whether the birth gestational age was preterm (birth gestational age < 37 weeks was preterm). Variables with statistical significance (*P* < 0.05) in the univariate analysis were tested for multicollinearity before being included in multivariate logistic regression analysis, and genes characterized by statistical significance (*P* < 0.05) in the multivariate analysis were considered as potential biomarkers of NS. The dataset was randomly split into a training set (70%) and a test set (30%). A logistic regression model was constructed on the training set, with NS potential biomarkers as independent variables. The diagnostic value of the model was evaluated on the training and test sets, respectively: decision boundaries were plotted to demonstrate the model's classification ability; using the “pROC” package (v1.18.5), the predictive power of the model was evaluated by plotting receiver operating characteristic (ROC) curves and calculating the area under the curve (AUC); using the “rms” package (v6.8.1), the reliability of the model predictions was assessed by performing an internal calibration with 1,000 resamples and plotting calibration curves.

#### 2.2.5 Immune infiltration analysis

CIBERSORT immune infiltration analysis was performed based on the gene expression data of all samples. Since CIBERSORT used a linear support vector regression model (28), and the downloaded gene expression data had been log-transformed, we applied exponential transformation to restore the original expression values. The infiltration matrix of 22 immune cell types was calculated using the “CIBERSORT” package (v0.1.0), and samples with *P* < 0.05 were screened. Differences (*P* < 0.05) in infiltration abundance between groups of immune cells were analyzed, and the correlation coefficient *R* between potential biomarkers and infiltration abundance of immune cells was calculated.

#### 2.2.6 Statistical analysis

Statistical analysis of the data was done in RStudio (v2023.12.1.402) using R language version 4.3.3. Differences between two groups were compared using the Mann–Whitney *U*-test, and correlations were analysed using the Spearman correlation coefficient, with a correlation coefficient >0.6 deeming the presence of a strong correlation. In the multicollinearity test, a tolerance >0.1 and variance inflation factor (VIF) < 10 were considered as no multicollinearity. *P* < 0.05 was considered as statistically significant difference.

To ensure reproducibility, a fixed random seed (seed = 1,234) was used for all procedures involving randomness (including the 70/30 train–test split, resampling-based calibration, and model fitting steps where applicable). The overall analytical workflow is summarized in Supplementary Figure S1.

## 3 Results

### 3.1 Concurrent innate immune activation and adaptive immune suppression

**Figure 2:**
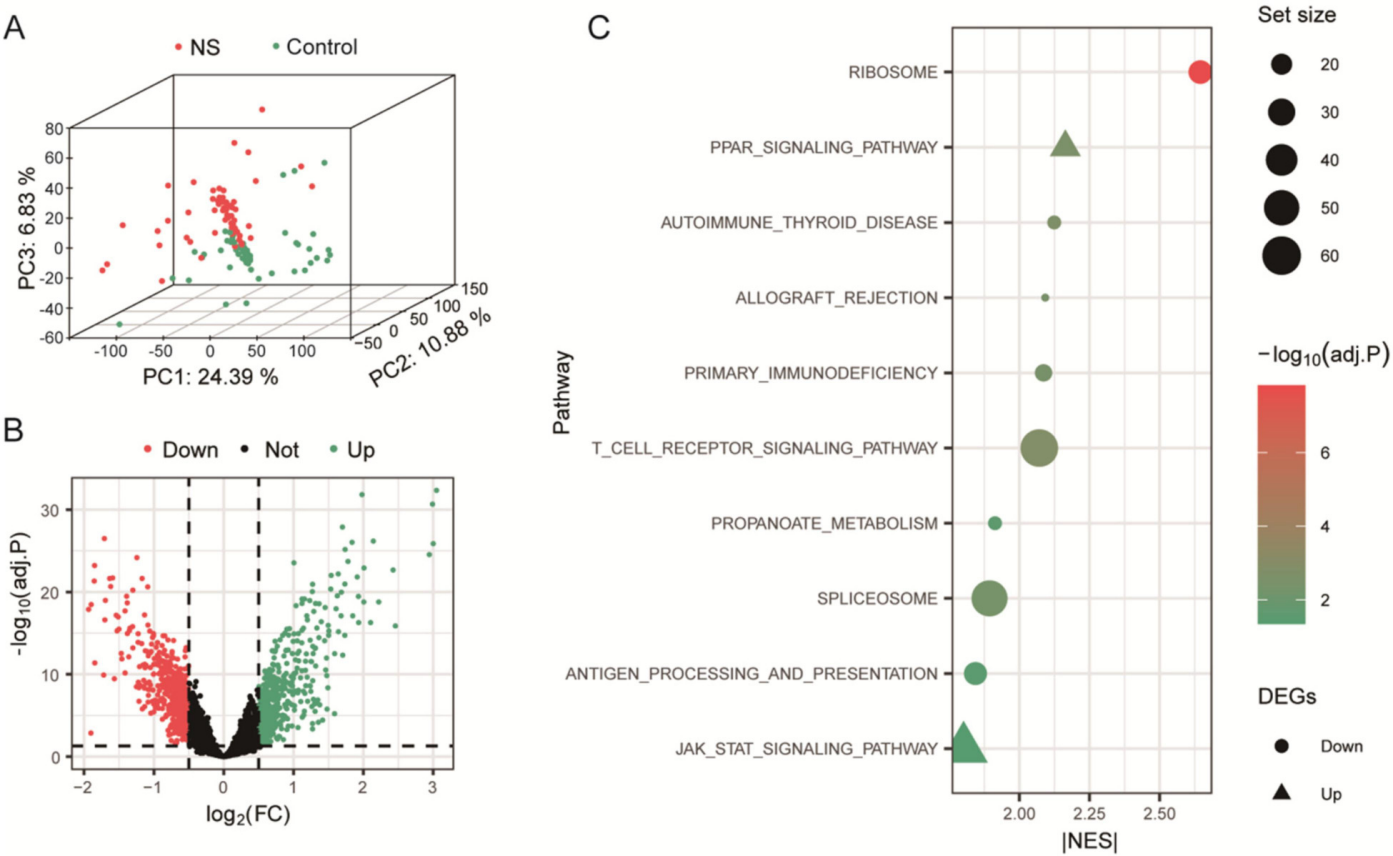
DEGs screening and GSEA significantly enriched pathways. **(A)** Three-dimensional principal component analysis (3D PCA) of global gene expression across all samples. Each point represents one sample, colored by its group. The first three principal components (PC1, PC2, PC3) explain 24.39%, 10.88%, and 6.83% of the total variance, respectively, indicating clear separation between groups. **(B)** Volcano plot of DEGs comparing NS vs. Control. Genes with a |log_2_FC| > 0.5 and an adjusted *P* < 0.05 are considered significant. Significantly down-regulated and up-regulated genes are highlighted in red and green, respectively, while non-significant genes are shown in black. **(C)** Plot of the significantly enriched pathways from GSEA, ranked by normalized enrichment score (NES). Pathways with an adjusted *P* < 0.05 were considered significant.

PCA result showed that NS samples were separated from control samples in the three-dimensional principal components of gene expression (Figure 2A). There were 992 DEGs after screening by threshold, of which 475 were up-regulated and 517 were down-regulated in expression (Figure 2B). The 10 biological pathways significantly enriched for a total of 129 core genes were obtained by GSEA (Figure 2C). The enriched pathways showed a clear bidirectional immune pattern in neonatal sepsis. Pathways related to pathogen sensing and innate inflammation were enriched in the sepsis group, whereas T-cell–associated pathways (e.g., T-cell receptor signaling) tended to be suppressed. This pattern is consistent with the concept that sepsis involves not only hyper-inflammation but also early and/or progressive adaptive immune dysfunction (immunosuppression/immunoparalysis).

### 3.2 Sepsis-associated WGCNA modules

**Figure 3:**
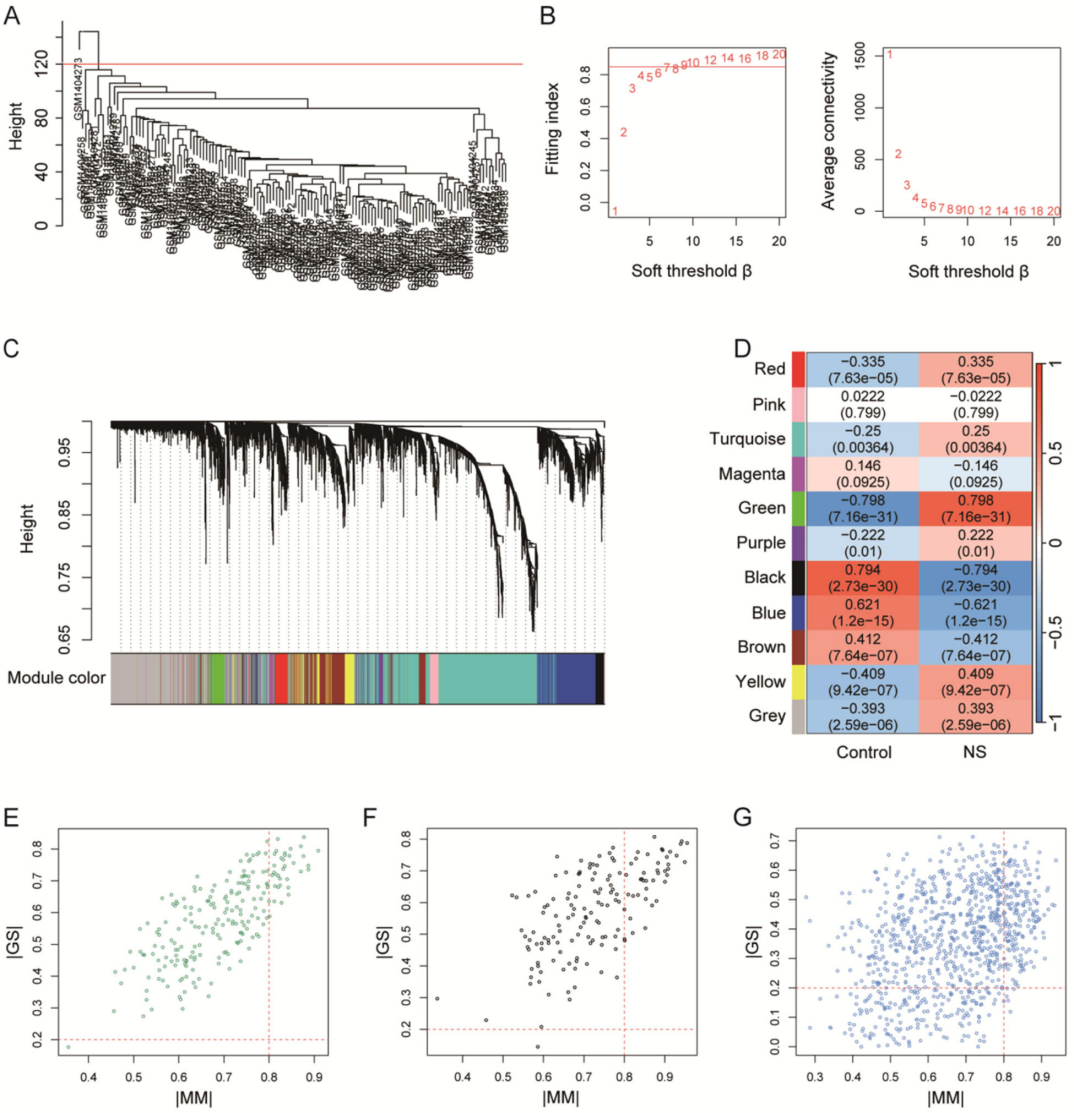
WGCNA identifies key gene modules associated with NS. **(A)** Sample clustering dendrogram based on global gene expression, used to detect outliers. One sample (above the red line) was identified as an outlier among the initial 135 samples and was excluded from subsequent network construction. **(B)** Analysis of network topology for various soft-thresholding powers (*β*). The left panel shows the scale-free fit index, and the right panel shows the mean connectivity. *β* = 9 was selected as the optimal value, where fit index first exceeded 0.85. **(C)** Hierarchical clustering dendrogram of genes and the resulting co-expression modules identified by WGCNA. Genes are clustered based on topological overlap, with each module assigned a unique color. **(D)** Module-trait associations. Each cell contains the eigengene significance (ES) value and its corresponding *P* (in parentheses) for the correlation between a module's eigengene and NS. The key modules—green, black, and blue—show the strong significant associations with NS. **(E–G)** Scatter plots of gene significance (GS) for NS vs. module membership (MM) in the **(E)** green, **(F)** black, and **(G)** blue modules, respectively. Genes with high intramodular connectivity (|MM| > 0.8) and strong association with NS (|GS| > 0.2) are considered key genes.

The result of hierarchical clustering showed that there was one outlier sample among 135 samples (Figure 3A), then the outlier sample was excluded. A soft threshold *β* = 9 was chosen, at which point the fitting index was >0.85 for first time and the change of the average connectivity was smooth (Figure 3B). The number of genes in the module was defined to be at least 30, and the threshold of modules merging was set to 0.25, resulting in the generation of 11 gene modules (Figure 3C). The results of the ES calculation for the modules (Figure 3D) showed that the key modules were green (|ES|=0.798, *P* < 0.001), black (|ES|=0.794, *P* < 0.001), and blue (|ES|=0.621, *P* < 0.001). The key genes in these three modules were filtered according to the criteria of |MM|>0.8 and |GS|>0.2 (Figures 3E–G), and 256 key genes were obtained in total.

### 3.3 Consensus ML feature selection

**Figure 4:**
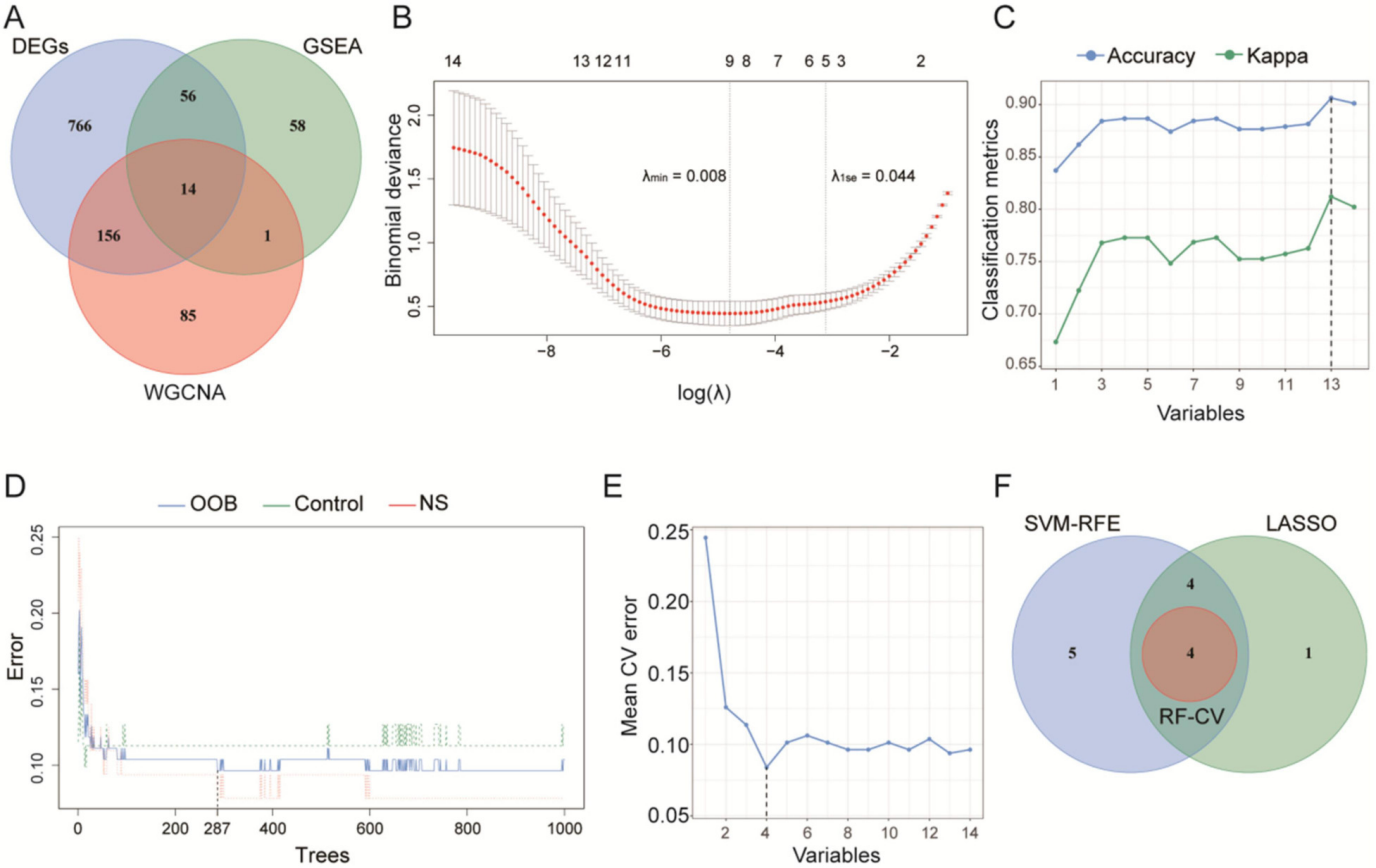
Integrative machine learning-based feature selection identifies a robust 4-gene signature for NS. **(A)** Venn diagram illustrating the overlap of candidate genes derived from three approaches: DEGs, GSEA, and WGCNA. The intersection yielded 14 consensus candidate genes for subsequent analysis. **(B)** Feature selection using LASSO. The left vertical dashed line indicates the optimal *λ* (*λ* = 0.008) at which the model achieves minimum cross-validation error, selecting 9 feature genes (non-zero coefficients). **(C)** Feature selection using SVM-RFE. A total of 13 feature genes were selected (indicated by a vertical dashed line) at the point where both accuracy and kappa were maximized. **(D)** Determination of the optimal number of trees (ntree) for the RF model. The OOB error stabilizes at a minimum when ntree = 287 (indicated by a vertical dashed line), which was chosen as the optimal parameter. **(E)** Feature selection using RF-CV. The model achieved the lowest mean cross-validated error with 4 genes (indicated by a vertical dashed line). **(F)** Final consensus feature selection. The Venn diagram shows the overlap of the feature genes independently selected by LASSO (9 genes), SVM-RFE (13 genes), and RF-CV (4 genes). The intersection of all three methods yielded a highly robust set of 4 core feature genes.

The intersection of the filtered DEGs, the core genes of GSEA, and the key genes of WGCNA yielded 14 overlapping genes (Figure 4A). Machine learning algorithms were applied to these 14 genes for feature selection, in which 9 feature genes were selected by LASSO regression (Figure 4B), 13 feature genes were selected by SVM-RFE (Figure 4C). In the Random Forest step, the final number of trees was 287 (selected based on the OOB error curve, Figure 4D), and 4 feature genes were selected through cross-validation (Figure 4E). Finally, 4 feature genes were obtained by taking the intersection of the three (Figure 4F): ACSL1, CD8B, CD3D, and JAK2.

### 3.4 Performance of the two-gene model

The results of the univariate logistic regression analysis showed that the coefficients of ACSL1, CD8B, CD3D, JAK2, birth weight, and birth gestational age were statistically significant, as shown in Table 1; the multicollinearity test showed that there was a small risk of high correlation between these variables, and so they were included in the multivariate logistic regression analysis. The final results showed that the coefficients of ACSL1 and CD3D were statistically significant. The results of the multicollinearity test and multivariate logistic regression analysis are shown in Table 2. We observed an extremely large and unstable odds ratio for antibiotic use in the univariate model, consistent with quasi-separation (antibiotics being administered predominantly after clinical suspicion/diagnosis). Therefore, we excluded this post-diagnosis intervention variable from the multivariate diagnostic model and explicitly acknowledge the instability of such clinical covariates in retrospective, small-sample settings.

**Table 1:** Results of univariate logistic regression analysis.

Variable*	Coefficient	SE*	Statistic	*P*	OR*	95% CI*
**ACSL1**	2.548	0.434	5.875	<0.001	12.782	[6.058, 33.852]
**CD8B**	−3.483	0.709	−4.913	<0.001	0.031	[0.006, 0.104]
**CD3D**	−2.800	0.487	−5.748	<0.001	0.061	[0.020, 0.140]
**JAK2**	3.996	0.709	5.640	<0.001	54.380	[15.516, 253.8]
**Birth weight**	2.817	0.495	5.696	<0.001	16.727	[6.761, 48.239]
**Birth gestational age**	2.554	0.431	5.920	<0.001	12.858	[5.716, 31.335]
Gender	−0.484	0.355	−1.364	0.173	0.616	[0.305, 1.232]
Antibiotics	19.428	1,118.623	0.017	0.986	2.738 × 10^8^	[0, 3.928 × 10¹⁵⁶]

SE, standard error; OR, odds ratio; CI, confidence interval.

Variables with *P* < 0.05 are highlighted in bold.

**Table 2:** Results of multivariate logistic regression analysis.

Variable*	Multicollinearity		Multivariate logistic regression					
	Tolerance	VIF	Coefficient	SE*	Statistic	*P*	OR*	95% CI*
**ACSL1**	0.566	1.766	1.529	0.683	2.239	0.025	4.614	[1.432, 21.568]
CD8B	0.481	2.080	−0.369	0.992	−0.372	0.710	0.691	[0.085, 4.558]
**CD3D**	0.395	2.531	−1.711	0.831	−2.058	0.040	0.181	[0.027, 0.791]
JAK2	0.700	1.429	1.528	0.921	1.658	0.097	4.609	[0.830, 34.378]
Birth weight	0.189	5.294	1.391	2.229	0.624	0.533	4.019	[0.067, 311.046]
Birth gestational age	0.189	5.290	2.202	2.099	1.049	0.294	9.043	[0.264, 577.669]

SE, standard error; OR, odds ratio; CI, confidence interval.

Variables with *P* < 0.05 are highlighted in bold.

**Figure 5:**
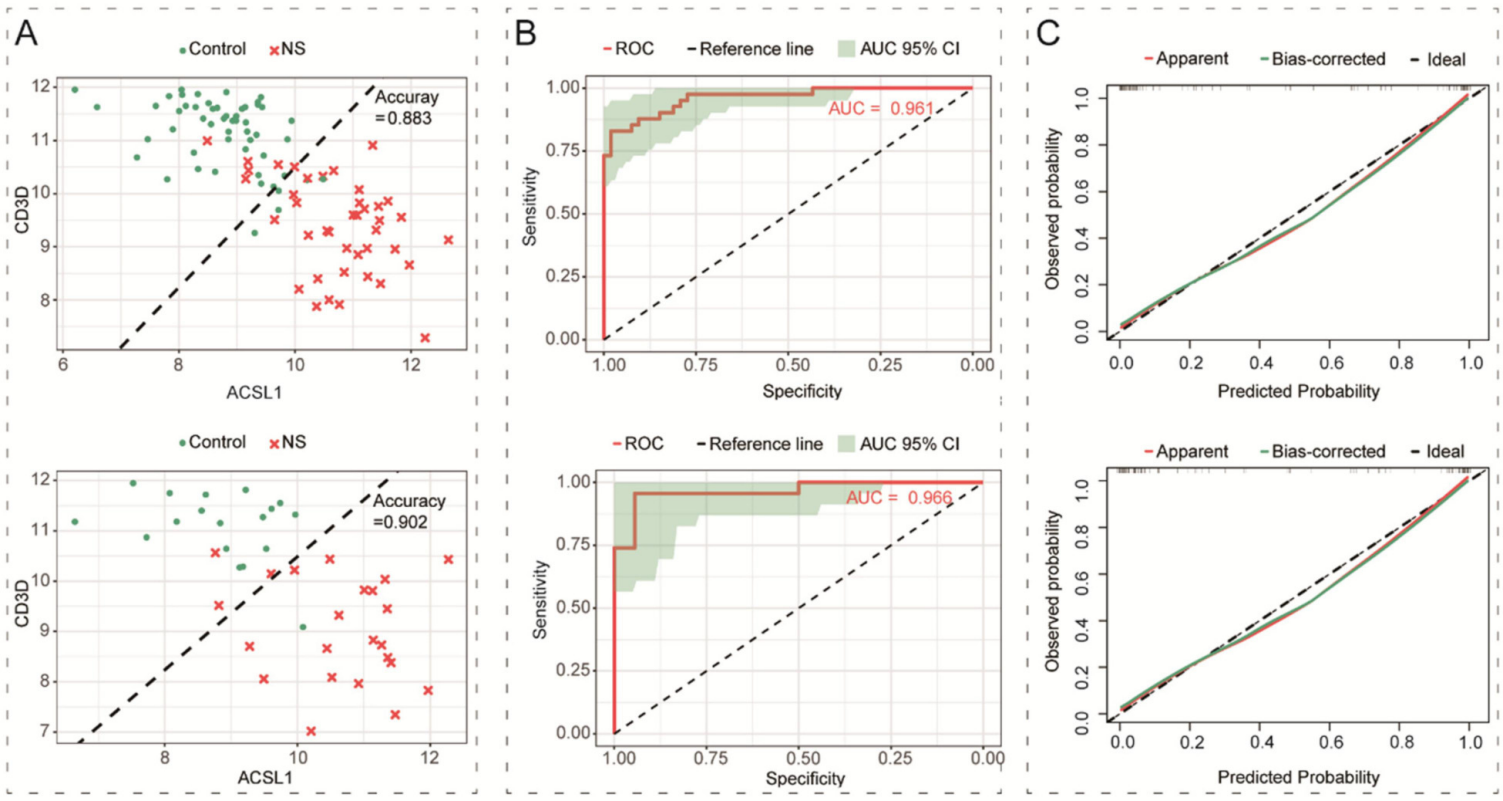
Evaluation of the model based on ACSL1 and CD3D expression. **(A)** Decision boundary analysis of the logistic regression model on the training set (top) and the test set (bottom). The dashed black line represents the decision boundary at a classification threshold of 0.5. The model achieved a classification accuracy of 88.3% on the training set and 90.2% on the test set. **(B)** ROC curves of the model on the training set (top) and the test set (bottom). The diagonal dashed line represents the performance of a random classifier (AUC = 0.5). The AUC was 0.961 for the training set and 0.966 for the test set. **(C)** Calibration curves for the training set (top) and the test set (bottom). The diagonal dashed line (ideal line) represents the ideal scenario where predicted probabilities perfectly match observed frequencies. The apparent calibration curve (red) was derived from the original dataset, while the bias-corrected curve (green) was obtained after bootstrap internal validation (1,000 repetitions). The close alignment of the model's bias-corrected curves to the ideal line across both datasets.

Using the potential NS biomarkers ACSL1 and CD3D as predictors, a logistic regression model was constructed on the training set. The model's output probabilities *p* were subsequently calculated for both the training and test sets. Taking a classification threshold of 0.5 as an example (i.e., diagnosing NS when *p* > 0.5), the decision boundaries on both the training and test sets showed that the model had good classification ability, with a classification accuracy of 0.883 on the training set and 0.902 on the test set (Figure 5A). ROC curves visualize the trade-off between sensitivity and specificity across all possible classification thresholds. The model demonstrated good predictive performance, with AUC values of 0.961 on the training set and 0.966 on the test set (Figure 5B). The calibration curves on both the training and test sets were essentially close to the ideal curve (Figure 5C), indicating that the model's prediction was reliable.

### 3.5 Immune-deconvolution trends

**Figure 6:**
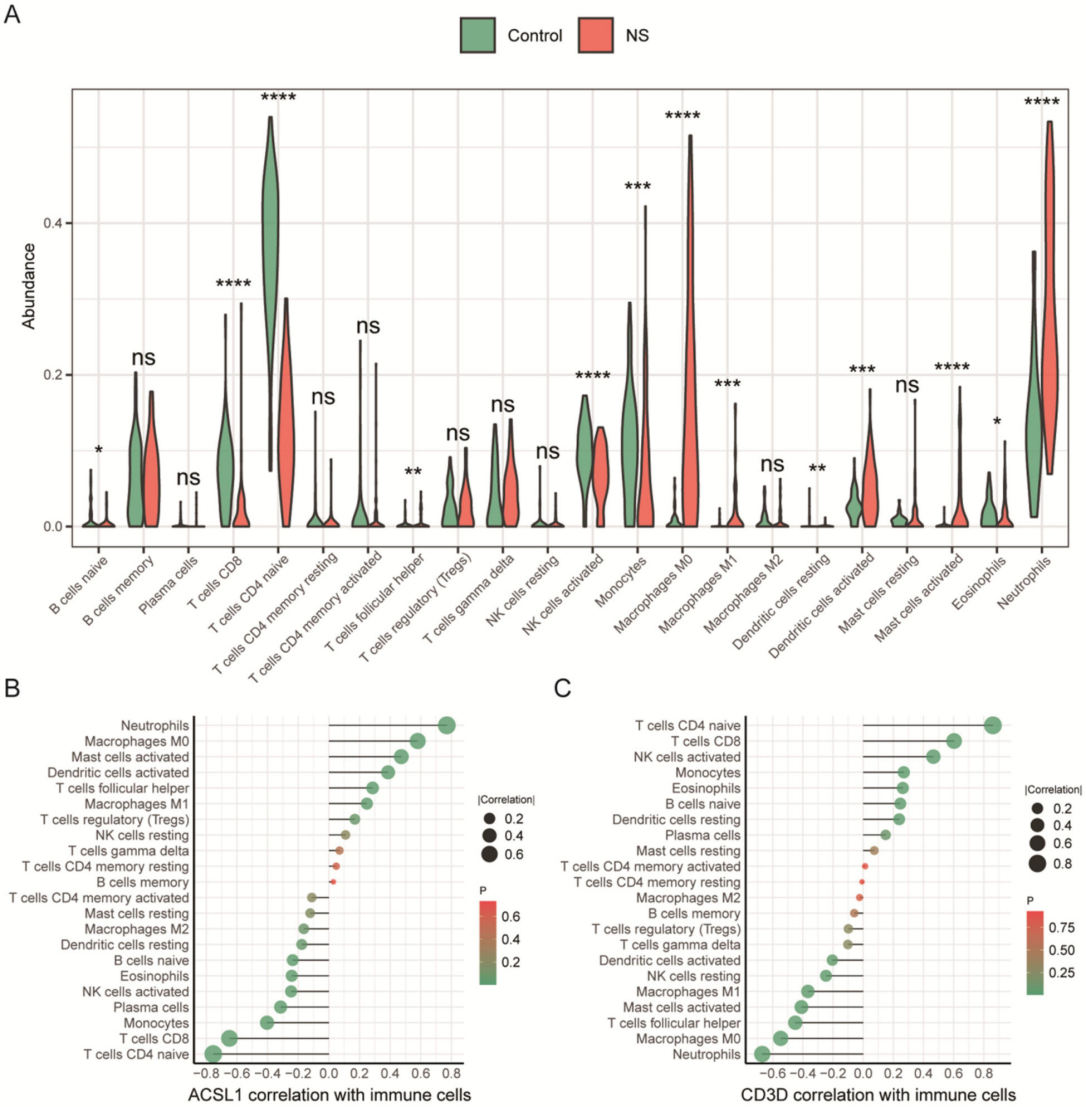
Differential immune infiltration landscape and its correlation with ACSL1/CD3D. **(A)** Violin plots comparing the infiltration abundance of 22 immune cell types between the NS and Control groups, as estimated by the CIBERSORT algorithm. Each plot shows the distribution (median, density) of immune cell fractions. Significance levels are indicated as: ns (*P* ≥ 0.05), * (*P* < 0.05), ** (*P* < 0.01), *** (*P* < 0.001), **** (*P* < 0.0001). **(B,C)** Scatter plots showing the correlation between the expression levels of biomarkers ACSL1/CD3D and the infiltration abundance of significantly altered immune cell subsets.

The results of CIBERSORT infiltration analysis were significant for all 135 samples. The infiltration abundance of 13 out of 22 immune cell types, including naive B cells, CD8^+^ T cells, etc., differed significantly between NS and control samples (Figure 6A). Because CIBERSORT relies on the LM22 reference signature matrix derived primarily from adult immune transcriptomes, we interpret the immune-deconvolution results as relative shifts/trends rather than exact quantification of neonatal immune-cell fractions.

The results of the correlation calculation (Figures 6B,C) showed that ACSL1 was positively correlated with neutrophils (*R* = 0.770, *P* < 0.001), and negatively correlated with naive CD4^+^ T cells (*R* = −0.754, *P* < 0.001), CD8^+^ T cells (*R* = −0.649, *P* < 0.001), and that CD3D was positively correlated with naive CD4^+^ T cells (*R* = 0.861, *P* < 0.001), CD8^+^ T cells (*R* = 0.603, *P* < 0.001), and negatively correlated with neutrophils (*R* = −0.670, *P* < 0.001).

## 4 Discussion

Neonates, being a vulnerable population with underdeveloped immune, skin, and mucosal barriers, are prone to sepsis and may swiftly advance to infectious toxic shock and multi-organ failure upon infection. The symptoms and signs of NS resemble those of premature newborns, complicating clinical diagnosis (7). To enable prompt and efficient clinical identification of NS cases, it is essential to analyze and identify potential biomarkers of NS.

This study screened 992 DEGs, determined 10 significantly enriched pathways comprising 129 core genes through GSEA, recognized 3 key modules totaling 256 key genes via WGCNA. Utilizing these three bioinformatics methods, 14 genes potentially associated with NS were isolated from a pool of 6,423 genes. The three machine learning algorithms were employed to select 4 feature genes from a set of 14 genes, then a “univariate followed by multivariate” logistic regression analysis was conducted. Ultimately, ACSL1 and CD3D were identified as potential biomarkers of NS. The CIBERSORT immune infiltration study indicated substantial variations in the abundance of 13 immune cell types, implying that various immunological mechanisms contribute to the pathogenesis of NS. ACSL1 showed strong correlations with neutrophils, naive CD4^+^ T cells, and CD8^+^ T cells, while CD3D exhibited similar associations but in the opposite directions.

ACSL1 and immunometabolic activation. ACSL1 encodes a long-chain acyl-CoA synthetase involved in fatty-acid activation and immunometabolic reprogramming (29). In bacterial sepsis, innate immune cells—particularly neutrophils/monocytes—undergo metabolic rewiring that supports rapid effector functions. The upregulation of ACSL1 in our analysis is consistent with heightened innate immune activation and may reflect a metabolically primed inflammatory state in neonatal sepsis. Numerous transcriptome datasets have shown that ACSL1 expression is markedly elevated in patient blood, neutrophils, and mice macrophages following bacterial infection and inflammatory stimuli (30, 31), corroborating the findings of the current work.

CD3D, adaptive immune suppression, and “immunoparalysis”. CD3D is a structural component of the T-cell receptor (TCR)/CD3 complex and is critical for T-cell activation signaling (32). Its downregulation, together with the observed suppression of T-cell–associated pathways (e.g., TCR signaling) and reduced T-cell–related signals in immune deconvolution, supports the concept that neonatal sepsis involves adaptive immune dysfunction (T-cell exhaustion/immune paralysis) in addition to hyper-inflammation. A study has demonstrated the evidence of T cell exhaustion in spleens from sepsis patients (33). Thus, CD3D is not merely a generic T-cell marker; it provides a mechanistic window into sepsis-related immune suppression that may have value for immune-status monitoring.

Comparison with existing biomarkers. Traditional biomarkers used in neonatal sepsis, such as CRP and procalcitonin, mainly capture non-specific acute inflammation and can be confounded by non-infectious conditions in neonates. In comparison with recently reported sepsis biomarker candidates, including targeting matrix metalloproteinase-9 (MMP9) to alleviate T cell exhaustion (34), a single-cell sequencing and machine learning-derived marker LILRA5 (35), B cell-derived ELL2 identified through multi-omics integration (36), and a monocyte differentiation-related signature developed via machine learning-based integration (37), our ACSL1 + CD3D pair possesses its own distinct advantages. It integrates two complementary dimensions—innate immunometabolic activation and adaptive immune suppression—potentially offering improved biological specificity.

Limitations and future validation. Several limitations warrant emphasis. First, the model was trained and tested within a single GEO dataset (GSE25504); even with a train/test split, this is not true external validation, and the reported AUC may represent an optimistic upper bound. Second, the sequential filtering strategy (DEGs → GSEA/WGCNA → ML → logistic regression) can introduce information leakage/optimism bias when candidate selection uses the full dataset; we therefore toned down claims and explicitly state this risk. Third, detailed clinical metadata (e.g., early- vs. late-onset sepsis and culture status) are not available in the public dataset, limiting phenotypic stratification. Fourth, antibiotic use is a post-diagnosis intervention and shows quasi-separation; such covariates are unstable in retrospective small cohorts. Finally, immune deconvolution based on adult-derived reference signatures (LM22) may be less accurate in neonates. Future work should include independent multi-center cohorts, platform-level replication, and wet-lab validation (e.g., qPCR) to confirm diagnostic and prognostic utility. Accordingly, our results should be interpreted as exploratory, hypothesis-generating biomarker discovery in a single public cohort, and diagnostic readiness will require validation across independent cohorts, platforms and institutions.

## Data Availability

The datasets presented in this study can be found in online repositories. The names of the repository/repositories and accession number(s) can be found in the article/Supplementary Material.
