# Supplementary material for: Bioinformatics combined with machine learning for potential biomarker screening and immune infiltration analysis in neonatal sepsis
Source: Front Pediatr. 2026 Mar 31;14:1702073. doi: 10.3389/fped.2026.1702073 (PMC13077849; doi:10.3389/fped.2026.1702073)
Supplement: Supplementary file 1 [file Image1.pdf]

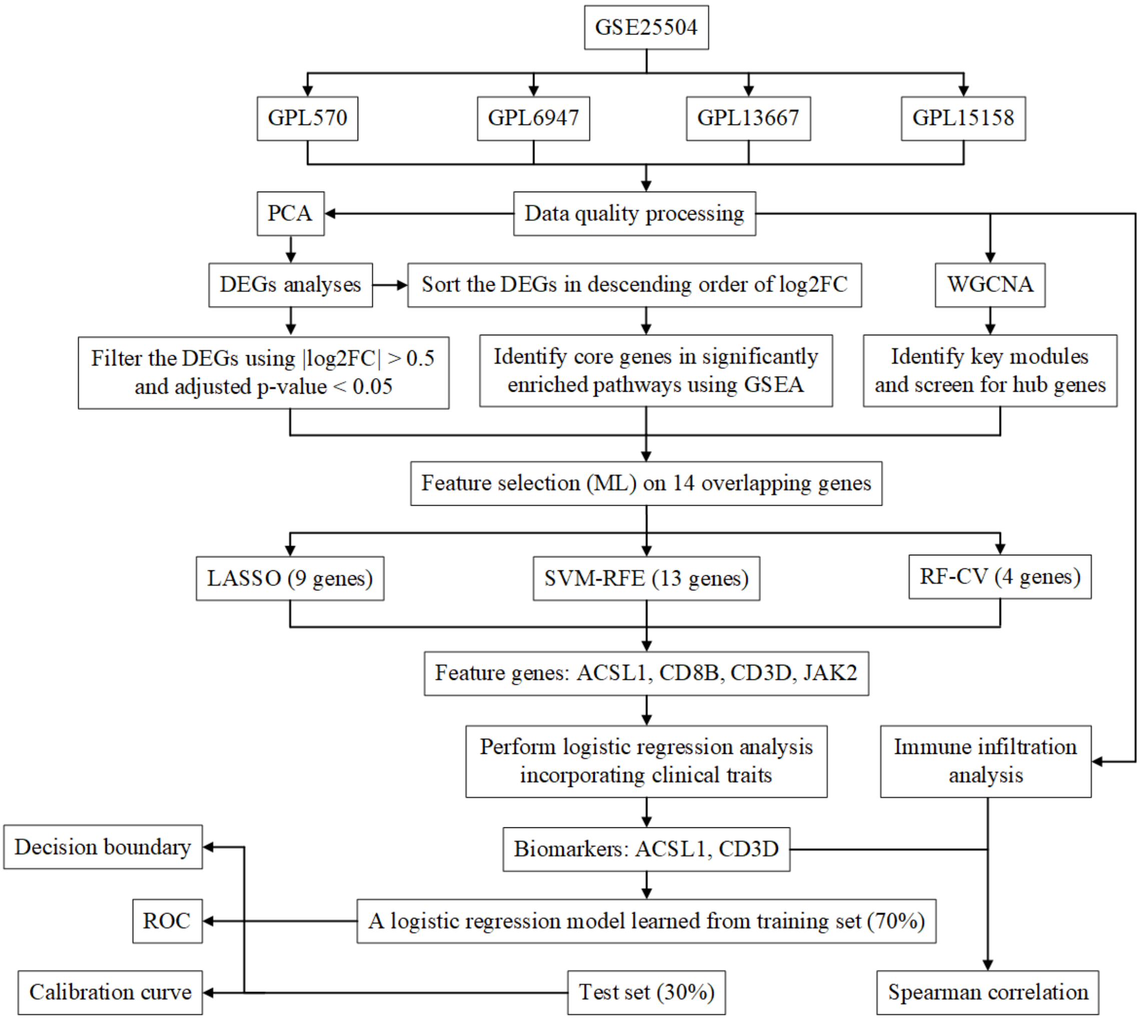

Supplementary Figure S1 Workflow diagram summarizing the analytical pipeline used in this study. This workflow provides an at-a-glance overview from data acquisition to biomarker discovery and immune deconvolution.
